# Prompt identification of struggling candidates in near peer-led basic life support training: piloting an online performance scoring system

**DOI:** 10.1186/s12909-023-04225-0

**Published:** 2023-05-02

**Authors:** Lawrence Gillam, Benjamin Crawshaw, Matthew Booker, Sarah Allsop

**Affiliations:** 1 Nottingham University Hospitals, Nottingham, UK; 2 Royal Berkshire Hospital, Reading, UK; 3 University of Bristol Medical School, Bristol, UK

**Keywords:** Peer teaching and assessment, Basic Life Support, Immediate Feedback, Scoring system, Competency Assessment Instrument

## Abstract

**Background:**

Bristol Medical School has adopted a near peer-led teaching approach to deliver Basic Life Support training to first year undergraduate medical students. Challenges arose when trying to identify early in the course which candidates were struggling with their learning, in sessions delivered to large cohorts. We developed and piloted a novel, online performance scoring system to better track and highlight candidate progress.

**Methods:**

During this pilot, a 10-point scale was used to evaluate candidate performance at six time-points during their training. The scores were collated and entered on an anonymised secure spreadsheet, which was conditionally formatted to provide a visual representation of the score. A One-Way ANOVA was performed on the scores and trends analysed during each course to review candidate trajectory. Descriptive statistics were assessed. Values are presented as mean scores with standard deviation (x̄±SD).

**Results:**

A significant linear trend was demonstrated (P < 0.001) for the progression of candidates over the course. The average session score increased from 4.61 ± 1.78 at the start to 7.92 ± 1.22 at the end of the final session. A threshold of less than 1SD below the mean was used to identify struggling candidates at any of the six given timepoints. This threshold enabled efficient highlighting of struggling candidates in real time.

**Conclusions:**

Although the system will be subject to further validation, our pilot has shown the use of a simple 10-point scoring system in combination with a visual representation of performance helps to identify struggling candidates earlier across large cohorts of students undertaking skills training such as Basic Life Support. This early identification enables effective and efficient remedial support.

## Background

### Basic Life Support training at the University of Bristol Medical School

Basic Life Support (BLS) training is a fundamental part of any healthcare provider course and successful implementation of the skills improves patient outcomes [1–5]. Generally, medical schools within the UK adopt a lecture-based approach followed by hands-on sessions to teach BLS [6–8]. Despite an initial improvement in performance, this approach can lead to poor retention of knowledge and a limited skillset when performing BLS in the following months to years [6, 9–11]. Therefore, an approach was desired that promoted longer term retention of cognitive and technical skills.

A prior review of the literature found that students typically respond positively to near peer-led teaching, rating the quality of teaching as ‘good’ or ‘excellent’. Furthermore, near peer-led teaching supports students to reach a high skill level, as demonstrated by a high first-time pass rate when completing their assessment [7].

In 2017, the University of Bristol Medical School undertook a curriculum review of the 5-year undergraduate medical programme, to create a new state of the art curriculum known as ‘MB21’. As part of the development of the year 1 curriculum, a new near peer-led BLS training scheme (Resuscitation Mentorship Development (RMD)) was introduced [12].

The RMD scheme was originally introduced at the University of Birmingham in 1995, [13] but had never been transferred fully to another higher education institution. The set-up of the Bristol RMD scheme mirrors that delivered in Birmingham which has previously been described in detail and validated in the literature [7, 14]. Working collaboratively, teams at the University of Birmingham and Bristol, successfully introduced the scheme during a 2-year pilot (2017–2019), which was overseen by the European Resuscitation Council (ERC). RMD Bristol became an autonomous ERC registered centre of BLS training in 2019. To our knowledge the centre at Bristol is only the second scheme in the United Kingdom to offer fully near peer-led BLS training to its medical undergraduates as an integrated part of the medical curriculum.

The RMD Bristol scheme is set up to offer both ERC BLS Provider training (known as BLS-P courses) and Basic Instructor training Courses (known as BIC) [5, 15–17]. This means as well as providing BLS training for year 1 students, the scheme offers students in years 2–5 the opportunity to become BLS instructors, providing valuable teaching qualification, experience and development of leadership and management skills [11].

### Basic instructor courses (BIC)

In line with ERC guidance, following completion of a BLS provider course, any student interested in becoming a BLS instructor can apply for BIC training. The BIC is run by the designated RMD Bristol ERC Course Director. Training is delivered by qualified ERC instructor Trainers (ITs) including senior medical students and student alumni, senior clinical academics, and educators.

Each year, RMD Bristol’s instructors’ course is run over 2-days, following the BIC curriculum from the ERC. During the BIC, instructors are educated in teaching techniques and given time to practice with peers. Additionally, instructors undertake a session on assessing learners’ performance. All RMD instructors who are due to teach on the BLS-P courses must attend this course annually. The majority of instructors are 2nd year medical students, with a small number of returning instructors from higher years who return as ‘senior student instructors’ and provide support to the new teachers, both with teaching and how our course runs. Note all new instructors must teach satisfactorily on a minimum of two subsequent BLS-P courses after the training weekend to be upgraded to a full ERC instructor [18].

### Bristol BLS-P Courses

The RMD Bristol scheme delivers BLS teaching to approximately 270 first year medical students per annum. At the time of this study, four BLS-P courses were run annually. Each course is four sessions run over 4 weeks: three are 2 × 1 h of teaching, (therefore, 6 teaching sessions for each candidate) and the fourth a short revision session followed by the assessment. Learning is further supported by an accompanying online learning package provided by the ERC through their online learning platform ‘CoSY’ [19].

All sessions follow the curriculum laid out by the ERC. In Week 1 (sessions 1 and 2), students are taught to perform CPR, in Week 2 (sessions 3 and 4) students are taught how to use an AED and performing CPR is recapped, and finally in Week 3 (session 5 and 6) students are taught basic first aid including foreign body airway obstruction. This ultimately leads to learning over several weeks, where information and skills are tested and recalled weekly: this method of active recall and spaced repetition with feedback from peers has been demonstrated to improve long term retention of knowledge and skill [11, 20–22].

**Fig. 1:**
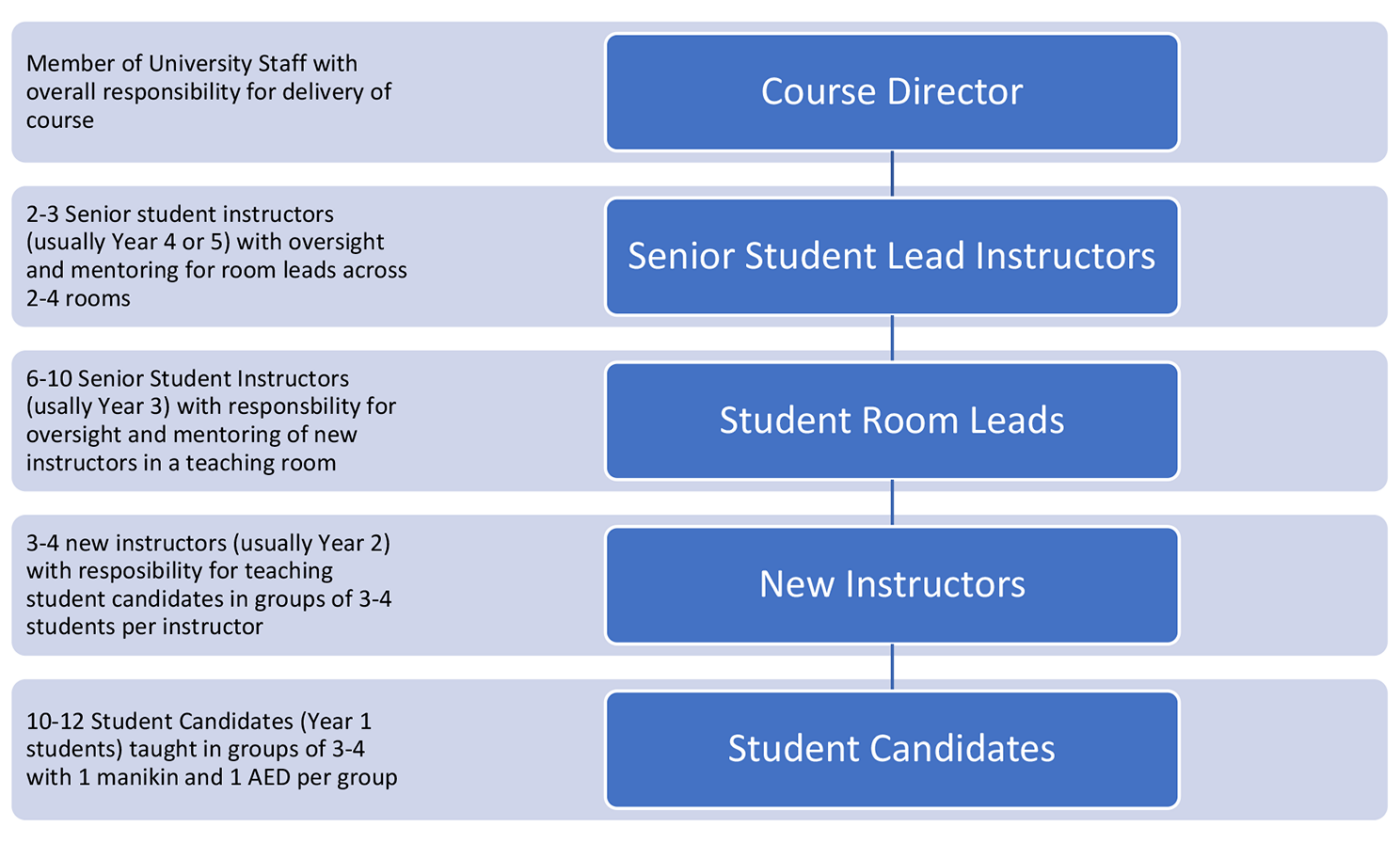
Outline of teaching structure for RMD Bristol BLS-P courses

Student instructors work in groups of 3–4 per room, supervised by a more senior student instructor who has been with the scheme at least 1 year to offer mentorship and support to new instructors, to teach 10–12 first year students. This gives a minimum instructor: candidate ratio of 1:4, in line with those recommended by the ERC [11]. The number of teaching rooms and instructors in any given course is varied to accommodate the total number of year 1 students in a given cohort, but typically BLS-P course run with 6–10 simultaneous rooms. 2–3 lead instructors and the course director have oversight of all rooms and offer support to the senior student instructors. An outline of the structure is provided in Fig. 1.

### BLS-P assessment

BLS-P assessment occurs in two ways, longitudinal formative assessment of candidate performance during the teaching sessions in weeks 1–3, and formal summative assessment of performance (in an exam setting) in week 4.

### Summative assessment

The formal assessment is undertaken as individual assessment of competency to deliver CPR, safely use an AED and put a casualty into the recovery position. This assessment is performed in a private room for each candidate, with 2 near-peer examiners using an examination template as a skills checklist, and an instructor acting as the ‘casualty’ for the recovery position. The validity of this near-peer assessment has been published by RMD Birmingham [7, 23]. Students who are unsuccessful at near-peer assessment are offered skills revision and then a resit assessment on the same night. Resit examinations are only performed by postgraduate staff members of the RMD scheme so that no student can fail another student. If a student fails the resit, they attend a further BLS-P course at a later date and take a third, and final, assessment completed by a senior staff examiner.

### Formative assessment and feedback to students

During the practice sessions, students would perform the skill being taught in front of their instructor and a small number of other students. Following the demonstration of the skill, the instructors would formatively assess their performance and provide feedback to the student utilising a ‘learning conversation’ style [24, 25]. Therefore, students were made aware of their performance with feedback immediately following demonstration of the skill.

### Supporting struggling student candidates

All student candidates undertaking BLS training at RMD Bristol are well supported through the instructors and the more senior, experienced student instructors who also act as leads in each room. Due to the increased training and experience of these more senior students, they are able to target candidates who are struggling in the development of new skills, if these individuals are brought to their attention by the new instructors. One of the challenges is how to help newer instructors identify and flag to more senior students which year 1 students need more support in a timely manner.

### “Traffic-light” scoring systems

Initially a system was introduced where instructors would highlight struggling candidates to the committee during session breaks and at the end of the session using a NEWS inspired [26] ‘traffic light’ system. The national early warning system (NEWS) is a system implemented in hospitals across the UK whereby a score is given to each of a patient’s vital signs (i.e. respiratory rate, blood pressure etc…) and then aggregated, with higher scores indicating more severely compromised physiology. This is often colour coded to provide a visual representation (green, amber, red) of effectively a 3-point scoring system.

At RMD Bristol, candidate performance was graded by their instructors, with those performing well or above expectations graded ‘Green’, candidates performing as expected were graded ‘Amber’ and candidates performing below expectations and therefore in need of additional support, were graded ‘Red’. However, senior student lead instructors were finding this did not differentiate enough between candidate abilities, and tended to identify those requiring additional support too late in the course to offer timely intervention and support. A further method of identifying these students more effectively was sought.

## Aims and objectives

Our objective was to develop and pilot an online scoring system in which a certain threshold could be used in order to more objectively identify candidates requiring additional support when taking part in BLS training. The aim was to integrate this system into the running of the course and allow immediate feedback to be acted upon in a timely and resource-efficient manner.

## Methods

### Numerical scoring systems

Senior students acting as room leads for the BLS-P courses found that they could better quantify the performance of student candidates subjectively by using a numerical scoring system, and scoring the observed skills out of 10. Having identified that some instructors were anecdotally using this 10-point scoring system to identify and quantify the performance of students, we wanted to review the use of this scale and investigate the value of formalising a system of recording this information, hoping to offer a consensus system for reporting student performance and in particular the identification of struggling students.

### Developing the consensus online scoring system ready for piloting

Following a period of discussion at teaching sessions and committee meetings, between the faculty of senior student instructors and staff, a 10-point scale was selected to pilot across all teaching sessions. This was selected over a 5-point, or 7-point scale to provide greater discriminating ability between student candidate performance, thus allowing for easier identification of excellent or struggling students.

Students were not informed of the score assigned to them, rather instructors provided formative verbal narrative feedback reflecting their performance.

Calibration was considered by asking senior student instructors to consider their scores when applied to exemplar candidate performances. Inter-rater calibration of assigned scores was based on the instructor’s experience at the training weekend when examples of sub-optimal BLS and AED use are shown. Intra-rater calibration across instructors was across both different groups of students within a teaching room, and then comparing this performance with that across the other teaching rooms. To improve internal consistency of scores, all instructors had to provide justification for score assignment, coupled with discussion from other instructors and senior faculty.

### Piloting the scoring system

In training sessions, instructors were asked to subjectively rate candidates from 1 to 10 via an online spreadsheet (Microsoft Excel, Version 16, Microsoft Corporation). Instructors were reminded that a score of 1 would represent the candidate being completely unable to perform the skill, and a score of 10 which would represent the candidate showing perfect demonstration of the skill. These scores were provided at the mid-session break and at the end of the teaching session and uploaded to the spreadsheet. Therefore, over the course each candidate would have a total of 6 scores. All student candidates follow the same pattern and order of the BLS training with the same instructor grouping each session. The two scores in week 1 (session 1&2) represents skills performance for the BLS algorithm (including CPR), two scores in week 2 (session 3&4) represent skills performance of adding the safe use of an AED to the algorithm, and two scores from week 3 (session 5) represents skills performance in first aid skills (including recovery position) and then a final score of BLS and AED use (session 6).

As previously stated, students were not informed of the score assigned to them, but feedback on their performance was given immediately following the demonstration of a given skill. For consistency, where possible, this score was provided by the same instructor each week with agreement from the room lead. If a candidate was taught by a different instructor, a score was still provided, with the room lead agreeing on the score to try and provide a degree of internal consistency. The internal consistency of the scoring is a result of all instructors undertaking the same training, with examples of good and poor performance with the addition of experienced room leads agreeing with or altering the assigned score.

The scores were inputted onto an online spreadsheet accessible to room leads, committee members and University faculty. A conditional formatting was used so that low numbers appeared red and high scores appeared green, and colours were graded: building on the initial ‘traffic light’ system.

### Data collection

The data, i.e. the scores assigned by instructors, was initially collected as a routine part of teaching sessions, and then inputted next to a person’s initials on an online spreadsheet, to allow simplified identification of students when cross referenced to the main candidate database (held separately as part of the external training package). No other demographics of candidates were recorded during this pilot. This scoring system was utilised in all 4 BLS provider courses for all first year medical students during academic year 2019-20.

### Threshold setting

In each course there were 3 committee members helping in the overall running of the course, in addition to the 6–10 room leads (Fig. 1). In each course there are between 65 and 75 students. Through experience it was found that when the number of students requiring additional support was more than 12 for a single week that this put considerable strain on resources. However, when the number of students requiring additional support was in the region of 8–10 per week this could be managed well. Therefore, it was concluded that identifying those in the lowest 15% of students would ensure that those in need of remedial support had the opportunity to get it without overwhelming the instructors and other committee members available. The decision to base this on historical evidence was also taken as the specific ability of each cohort could differ making them more, or less able when performing BLS.

### Analysis

In this retrospective analysis of data collected as part of teaching sessions: IBM SPSS version 27 was used to analyse the data. Tables were created using Microsoft Word (Version 16, Microsoft Corporation) and graphs were created using Microsoft Excel (Version 16, Microsoft Corporation). Descriptive statistics for each session, as well as the difference in scores between sessions was assessed. Normality of residuals for trend of data was checked. It was felt that a parametric test could be utilised, as per the work of Geoff Norman [27]. Interactions were checked. An Analysis of Variance (ANOVA) was used for the analysis of the scores over the first three courses to assess the difference between sessions of all three cohorts. Statistical significance was set at P < 0.05. This analysis of the first three cohorts was then used to set thresholds for highlighting students which was then applied to the final cohort to determine if students were more easily identified.

## Results

Overall, in this analysis there were 208 candidates over 3 cohorts of students from September 2019 to February 2020. Unfortunately, not all scores were entered by instructors for all students for all sessions, therefore some data points are missing from the analysis. The conditionally formatted spreadsheet gave a clear visual representation of a candidate’s performance, and instructors agreed that it was best utilised for visualising candidates scoring low. There was consensus among instructors that scores of less than 3 and 4 were most easily identified using the colour coded system, but as student’s performance improved this identifiable colour was lost, making it harder to identify potentially struggling students.

**Fig. 2:**
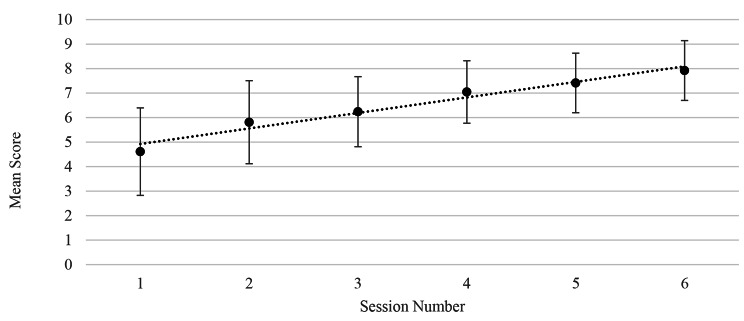
Mean scores ± standard deviation over the six session of the course demonstrating significant linear trend using a one-way ANOVA (P < 0.05). In session 1 and 2 CPR was covered, in session 3 and 4 AED use was covered and CPR skills recapped. Finally, in session 5 and 6 basic first aid skills were covered in addition to revising AED use and CPR

A significant linear trend in scores was identified using a one-way ANOVA over the course with mean scores from each session of 4.61, 5.81, 6.24, 7.05, 7.41 and 7.92 (Table 1: Fig. 2), with an associated P value < 0.05. A normal distribution of residuals from the linear regression model was confirmed, and no interaction based on a candidate’s room allocation or cohort detected.

**Fig. 3:**
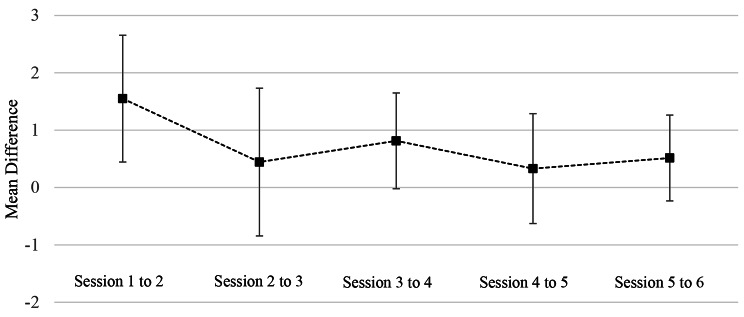
Mean difference between consecutive sessions ± standard deviation

The mean difference between consecutive sessions can be seen in Table 2; Fig. 3: generally small improvement could be seen throughout the course, with the largest increase seen between the 1st and 2nd session, implying that the greatest learning curve occurs in the initial phase of training. Although this 1st difference between sessions was larger than subsequent differences, there was no statistically significant difference among all the differences. No significant trend was identified.

There is a discrepancy between the differences between sessions seen in Fig. 2; Table 1 when compared to Fig. 3; Table 2, this may be as a consequence of missing data points in the data set. For the data to be analysed and presented in Fig. 3; Table 2 a scores for individual students had to be present for both sessions to calculate the difference. When a data point was missing the calculation and analysis could not be done.

**Table 1:** Mean score for each session over the first 3 courses taught in the year. In session 1 and 2 CPR was covered, in session 3 and 4 AED use was covered and CPR skills recapped. Finally, in session 5 and 6 basic first aid skills were covered in addition to revising AED use and CPR (Note the discrepancy in ‘n’ is due to missing instructor scores during data collection)

		*Session*	*Mean Score ± SD*
Week 1 *(BLS algorithm incl CPR)*	Mid-point	1	4.61 ± 1.78 (n = 149)
	End	2	5.81 ± 1.69 (n = 207)
Week 2 *(BLS plus AED use)*	Mid-point	3	6.24 ± 1.43 (n = 199)
	End	4	7.05 ± 1.27 (n = 200)
Week 3 *(Basic first aid and revision of BLS and AED)*	Mid-point	5	7.41 ± 1.22 (n = 162)
	End	6	7.92 ± 1.22 (n = 203)
		P < 0.05 for Linear Trend	

**Table 2:** Mean difference in scores between consecutive sessions ± Standard Deviation. The number of students were a value could be generated is reflected through ‘n’. (Note the discrepancy in ‘n’ between sessions is a consequence of missing data points)

	*Mean Difference in Score ± SD*
From Session 1 to 2	1.55 ± 1.11 (n = 149)
From Session 2 to 3	0.44 ± 1.29 (n = 198)
From Session 3 to 4	0.81 ± 0.84 (n = 199)
From Session 4 to 5	0.33 ± 0.96 (n = 159)
From Session 5 to 6	0.52 ± 0.75 (n = 162)

### Threshold setting

In an aim to continually highlight approximately 10 students (~ 15%) per session in each cohort that may require additional support, a decision to use the threshold historical cohort mean score minus 1 standard deviation was made. This decision was taken as, in normally distributed data, this would identify candidates scoring in the lowest 16% of the student population in total^1^. Adding to the ability to highlight candidates earlier is the utility of difference in scores between sessions, again the historical cohorts mean minus one standard deviation was used as a threshold below which candidates would be highlighted as potentially struggling candidates. The thresholds for each session are given in Table 3.

**Table 3:** Scores at which candidates would be highlighted by the conditionally formatted spreadsheet, which are equal to Mean-1SD, derived from the first 3 cohorts of students. In session 1 and 2 CPR was covered, in session 3 and 4 AED use was covered and CPR skills recapped. Finally, in session 5 and 6 basic first aid skills were covered in addition to revising AED use and CPR

*Session*	*Score*	*Difference in Score between Sessions*
1	< 2.8	-
2	< 4.1	< 0.4
3	< 4.8	< -0.8
4	< 5.8	< 0.0
5	< 6.2	< -0.6
6	< 6.7	< -0.2

Prior to the fourth course of the year starting, the conditional formatting of the scoring system was altered to reflect the historical mean ± SD for each session, and difference in score between sessions. As a result, the data was split into 4 groups:

1. Candidates scoring less than the (Mean – 1SD).
2. Candidates scoring between the Mean and (Mean – 1SD).
3. Candidates scoring between the Mean and (Mean + 1SD).
4. Candidates scoring greater than the (Mean + 1SD).

**Fig. 4:**
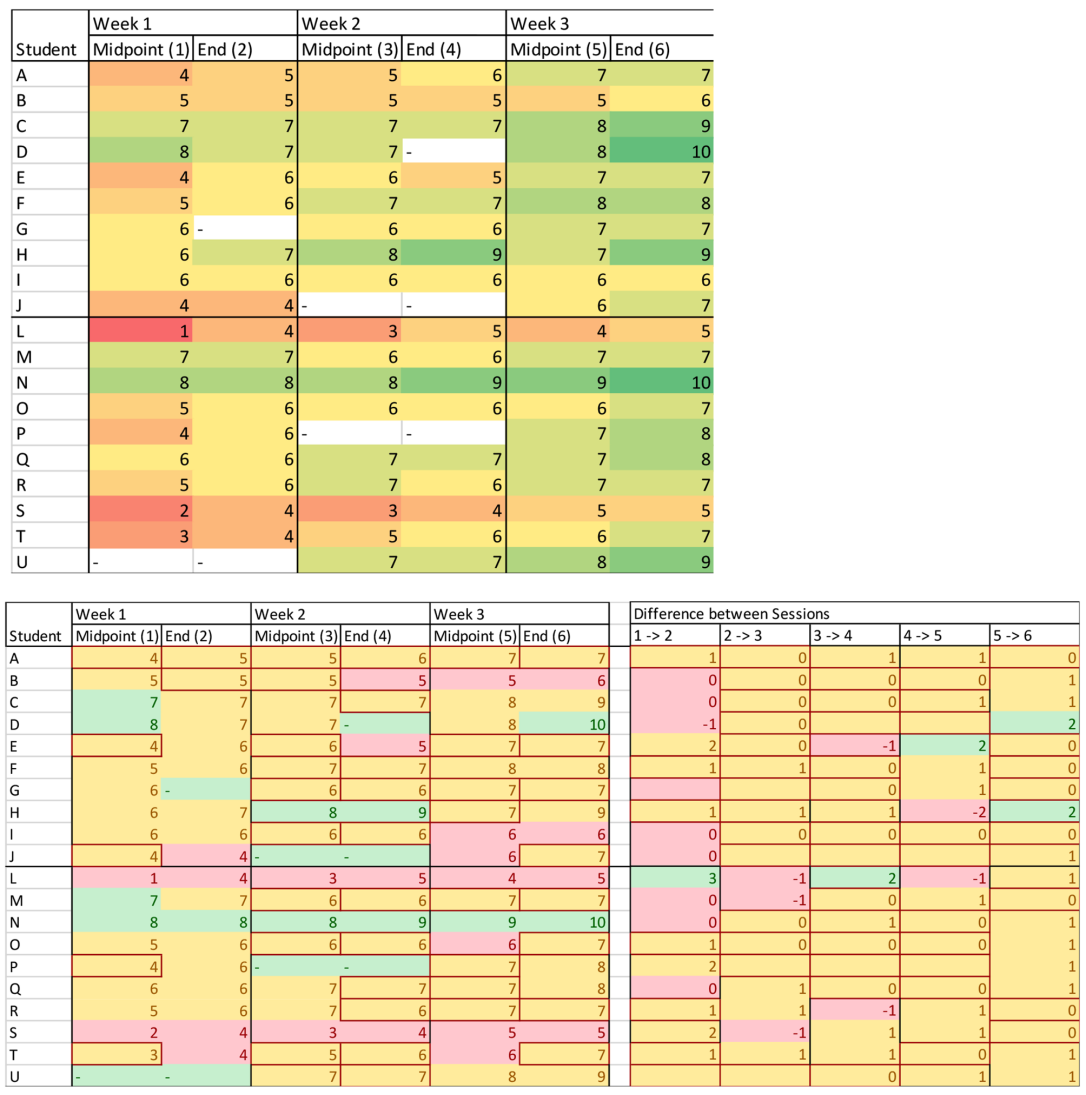
This is an example set with the same numbers used in the set above and below. *Above*: example of formatting before updated conditional formatting. Colours generally changes from shades of red to green over the course. Numbers of 4 or less are generally highlighted more easily with the darker shade of red, therefore are most easily identified by instructors and course leaders. As the course progresses there is less of this colour and therefore few candidates are highlighted. *Below*: the same dataset with updated example of conditional formatting. The scores given during each session are provided, as well as the difference in scores. Both ratings can be used to highlight struggling candidates, either individually or with each other. This new system continues to highlight candidates that may be struggling throughout the course that may have been missed when solely using the original system

The allocation of score by the instructor did not alter, but the conditional formatting used within the spreadsheet was altered in order to highlight candidates falling below the historical mean-1SD more easily. An example of the same scores before and after this change can be seen in Fig. 4.

## Discussion

By implementing a live feedback system within the delivery of near peer-led teaching to 1st year medical students this RMD Bristol pilot has been able to highlight struggling candidates with greater ease and offer remedial support more quickly without overwhelming course instructors.

Despite a lack of discussion in the literature about use of 10-point scale scoring systems within medical education, comparison can be made to an Objective Structured Clinical Examination (OSCE) style assessment whereby a checklist is primarily used in conjunction with a global rating scale [28]; typically a Likert scale. However, it is recognised that there is significant academic debate about the utility, appropriateness, and statistical handling of numerical scoring systems with different scales [27, 29], with many different soring systems available and different scales used such as 5-, 7- and 10-point scales, versus Likert-style agreement scales and visual score representations. There is much discussion in the literature of the relative merits of shorter and longer scale scoring systems, and the drawbacks of each [30, 31]. However, instructors’ preference was to use a 10-point scale due to its perceived familiarity and greater degree of discrimination it provided in comparison to a 5-, or 7-point scale. It is accepted that at present more work needs to be undertaken in order to fully assess the internal validity as well as the intra and inter-instructor rating. This is an area of planned future work. Despite this limitation, the scoring system does successfully achieve its aim at identifying students who are potentially struggling with their learning whilst not overwhelming instructors.

Many candidates initially have a score of less than 3 or 4, which is most easily recognised by the darker red colour it is assigned. Candidates improve over the course (Fig. 2) and the easily identifiable colour is lost. Given the limited number of instructors available to provide additional help to those struggling candidates, e.g. provide one-to-one teaching, it was felt that up to 10 candidates per week could be helped in this manner so as not to overload instructors. As a result, a method of identifying candidates that continued to highlight candidates scoring low within the cohort at a constant rate needed to be identified. Our utility of the mean score minus one standard deviation as a threshold was successful in continuing to identify potentially struggling students.

By utilising the difference in scores between sessions, those candidates that are not progressing as expected can be identified and instructors can intervene to support them. This additional method of highlighting candidates can be used in conjunction with how the candidate is performing: i.e. a candidate performing well, and deemed to be exceptional by their instructor and is scored 10 for every session will be highlighted as not showing any improvement and flagged by the spreadsheet, but would not require additional support. Where this system of highlighting the difference in score is particularly useful, is for candidates that are scoring around the average: for example, a *Student I* in Fig. 4 that scores 6,6,6,6,6,6 over the six sessions. In this example by using the score provided by the instructor *only*, they would be highlighted as struggling in session 5 (the last week of the course). However, by using the difference in score, this candidate would be highlighted as struggling/not improving by session 2 (the end of week 1). Therefore, the instructors would be aware to keep a look out for how the candidate is getting on.

By implementing this system, whereby, a ‘live’ conditionally formatted spreadsheet that is available to view by room leads and faculty, struggling candidates are identified quickly. At RMD Bristol, room leads gather together both during the mid-session break and at the end of the teaching session to discuss how candidates are performing; it is at this point which a score is assigned. As soon as a value has been inputted, a member of the senior committee or faculty overseeing the teaching evening then has a visual representation of how all candidates are performing and where to best allocate additional teaching resources (if required). As a result, struggling candidates are given remedial support immediately after that break, or are highlighted as requiring further help at the start of the next week. Instructors commented that the system was very easy to use, finding the immediacy of remedial support for their candidates reassuring.

As commented by *Li et al.*, struggling medical students is an under-researched area, despite it being acknowledged that early identification is key for ‘*effective remediation’*[32]. They were specifically commenting on students struggling with the course as a whole and determining if pre-admission tests were useful tools to identify ‘strugglers’ as they note that there is a need for ‘*effective tools and methods to accurately identify them’*. However, the idea is not too dissimilar to the issue faced at RMD Bristol. Furthermore, given that the BLS-P is delivered during a student’s first year of medical school it is not surprising to identify ‘strugglers’ given several potential factors in a period that *Picton et al.*, labelled a *‘critical transition’* [33]. At this point in a student’s journey there is a balance of numerous identities: the University Student who is developing independence, the Medical Student who is now a part of a competitive environment, in a large cohort asked to conduct more self-directed learning and the Doctor-to-be who is forming an early professional identity and learning to cope with pressure and sometimes failure. It has been noted that when those struggling with their learning are not supported early that there is an increased risk of discouragement, exhaustion and potential drop-out from medical school [33, 34]. The issue is that much of the remedial support provided at medical schools occurs once a problem has been identified, either due to academic failure, or another reason [35]. At RMD Bristol we have developed and piloted a tool that identifies struggling students earlier in their learning, prior to assessment, and consequently are able to provide remedial support in a timely manner. Our hope is that this input gives students improved skills, confidence and reduces the chance of failure on the course.

One of the major advantages of using this system is that a consistent number of candidates are highlighted week-on-week despite overall improvement in skill level. Therefore, instructors and committee members have a manageable number of candidates that they can provide additional support to. It should be noted that using this system should highlight the bottom 16% of the student population, therefore if by chance there is a cohort that is particularly good at BLS and AED use and are scored more highly by the instructors, they would not be highlighted as they would be performing above the threshold set. Therefore, no additional support would be required by this cohort.

Finally, with each passing course and further data collection the estimate of mean score can be continually updated and can help improve the precision of the threshold used to highlight candidates requiring remedial support. This makes any large differences between cohorts less notable when trying to highlight candidates that are struggling.

### Potential transferability of the scoring system

This system of live feedback could be implemented in a wide variety of educational institutions or courses. Ultimately, the idea condenses to two key transferable ideas: immediacy and threshold setting. Such a system can only be utilised if the input from instructors/teachers is used by senior members of faculty in a timely fashion, in our case this was within minutes. But this is contextual, in other courses or institutions this could take hours, days or weeks. Further to this point, the speed at which instructors can input their score aids rapid identification of struggling candidates. Therefore, a system needs to be in place to facilitate this input from instructors. The threshold setting at RMD was based on historical data of scoring from instructors, however, the threshold to highlight candidates could initially be set up based on curriculum goals, minimally acceptable performance, or several other metrics. As in our system, this threshold can continue to raise through the educational period in order to continually identify weaker candidates or students and be adapted based on previous cohorts if needed. As a pilot, although this system is yet to be validated, our hope is that the ideas presented could have a greater reach than just in life-support training.

### Limitations

At the time of this pilot, the use of this relatively simple, subjective 10-point scoring system is certainly not without its limitations, including: the score itself, the validity of the score and the incomplete dataset. Future work is needed before this scoring system can be rolled out and recommended.

The ‘score’ assigned to candidates by the instructors is subjective and relative; as inherent with most rating scales [28]. However, although this scoring system is subjective, there is a relative degree of internal validity. Each instructor must have successfully completed the instructor training course, an element of which is a session on how to assess. The instructors also have a conceptual framework on which to base their scores, so have a degree of anchoring. Finally, the instructors have several candidates they teach during the course and observe what other instructors score each candidate. However, formal descriptions are yet to be provided to instructors in order to help guide the score they provide. Work is underway to standardise descriptors for each assessment domain.

Following on from the previous point, the scoring system is yet to be externally validated. There is no external ‘gold standard’ of ability provided by the course providers and therefore the scoring system that has been developed cannot be compared to a standard. However, that is not to say that frameworks such as those proposed by Messick or Kane, or reviewed by Cook et al., [36–38], could not be implemented in order to validate this scoring system for use in near peer-led BLS training.

Demographic data was not collected as part of the current study as the focus was on the development of the scoring tool. It is acknowledged as a limitation of this work, and a potential extension to the work during the future use of the scoring system to further explore if there are any patterns to which students are more likely to struggling based on this type of data.

Finally, the optimal analysis for this work would have been a repeated measures ANOVA approach. However, this was not undertaken due to a number of missing data points across the set. This missing data could have been due to a single data point being missing, or multiple, ultimately with the same consequence. There are multiple reasons that this could be due to, including: candidate illness, instructors forgetting to upload a score, or instructors being unable to upload a score to name a few. Being unable to perform this analysis has likely limited the statistical power.

### Future work

Further work could be undertaken in order to strengthen the objectivity and consistency of our scoring system. This could then be used to validate this work. As there are other centres in the UK teaching these courses, it is possible that this scoring system could be used at alternative locations to determine if it is a useful method to highlight struggling candidates at other course centres.

At RMD Bristol the manikins used can provide ‘real-time’ feedback to the candidates about their depth, number and rate of chest compressions in addition to success and quality of ventilatory breaths. There is a feature whereby candidates perform a few cycles of CPR and a percentage score is given to say how effective their CPR and ventilatory breaths are being: termed Quality Cardiopulmonary Resuscitation (QCPR®) [39]. There has been previous work looking at this as a teaching tool and it has been demonstrated in a previous cluster randomised controlled trial and review that its use helps to improve participants BLS skills [40, 41]. Therefore, future work could involve using this QCPR percentage score to give a more objective way of scoring candidates and could be used to help validate the scoring system. However, this would not include all aspects of the candidate’s performance.

This work could ultimately be paired with how the candidates perform in their end of course assessment. This would help to provide a greater predictive value and highlight candidates most likely to struggle or fail the assessment. This work could also seek to collect demographic data about candidate performance to review if particular sub-groups of students are more likely to struggle and thus modified or additional support be offered for these groups. One could further examine the effect of being highlighted by our system and whether the candidate continues to pass the assessment, and whether this is done on first or second attempt.

Finally, future qualitative work could be done to examine how candidates that are highlighted by the scoring system find the course, and whether additional support is helpful. Qualitative work could also examine instructors’ thoughts around use of the scoring system to help determine its ease of use and perceived utility.

## Conclusion

Our pilot using a data driven ‘real-time’ conditionally formatted scoring system has shown potential as a tool for instructors and faculty to rapidly highlight candidates that are struggling on a practical skills-based Basic Life Support course. By highlighting these candidates more easily and more effectively they can receive additional support sooner and the hope is that these candidates feel more confident prior to their assessment and future work as clinicians.

## Data Availability

An initial analysis and summary of this work was presented as an e-poster at AMEE Virtual Conference 2020. Raw data and/or analysis can be made available on reasonable request to the corresponding author.

## Abbreviations

AED: Automated External Defibrillator.
ANOVA: Analysis of Variance.
BIC: Basic Instructor Course.
BLS: Basic Life Support.
BLS-I: Basic Life Support Instructor.
BLS-P: Basic Life Support Provider.
CPR: Cardiopulmonary Resuscitation.
ERC: European Resuscitation Council.
OSCE: Objective Structured Clinical Exam.
QCPR: Quality Cardiopulmonary Resuscitation.
RMD: Resuscitation Mentorship Development.
SD: Standard Deviation.

## Footnotes

1 Assuming normally distributed data, 68% of the data would fall between the Mean ± 1SD. Half of the remaining data would be below the Mean-1SD; i.e. 16%.
